# Characteristics of oral methicillin-resistant *Staphylococcus epidermidis* isolated from dental plaque

**DOI:** 10.1038/s41368-020-0079-5

**Published:** 2020-05-09

**Authors:** Boyu Tang, Tao Gong, Yujia Cui, Lingyun Wang, Chao He, Miao Lu, Jiamin Chen, Meiling Jing, Anqi Zhang, Yuqing Li

**Affiliations:** 1 State Key Laboratory of Oral Diseases & National Clinical Research Center for Oral Diseases & West China Hospital of Stomatology, Sichuan University, Chengdu, China; 2 Division of Infectious Diseases, Boston Children’s Hospital, Harvard Medical School, Boston, MA USA; 3 Department of Laboratory Medicine, West China Hospital, Sichuan University, Chengdu, China

**Keywords:** Clinical microbiology, Biofilms

## Abstract

The oral microbial community is widely regarded as a latent reservoir of antibiotic resistance genes. This study assessed the molecular epidemiology, susceptibility profile, and resistance mechanisms of 35 methicillin-resistant *Staphylococcus epidermidis* (MRSE) strains isolated from the dental plaque of a healthy human population. Broth microdilution minimum inhibitory concentrations (MICs) revealed that all the isolates were nonsusceptible to oxacillin and penicillin G. Most of them were also resistant to trimethoprim (65.7%) and erythromycin (54.3%). The resistance to multiple antibiotics was found to be largely due to the acquisition of plasmid-borne genes. The *mecA* and *dfrA* genes were found in all the isolates, mostly *dfrG* (80%), *aacA-aphD* (20%), *aadD* (28.6%), *aphA3* (22.9%), *msrA* (5.7%), and the *ermC* gene (14.3%). Classical mutational mechanisms found in these isolates were mainly efflux pumps such as *qacA* (31.4%), *qacC* (25.7%), *tetK* (17.1%), and *norA* (8.6%). Multilocus sequence type analysis revealed that sequence type 59 (ST59) strains comprised 71.43% of the typed isolates, and the eBURST algorithm clustered STs into the clonal complex 2-II(CC2-II). The staphyloccoccal cassette chromosome *mec* (SCC*mec*) type results showed that 25 (71.43%) were assigned to type IV. Moreover, 88.66% of the isolates were found to harbor six or more biofilm-associated genes. The *aap*, *atlE*, *embp*, *sdrF*, and *IS256* genes were detected in all 35 isolates. This research demonstrates that biofilm-positive multiple-antibiotic-resistant ST59-SCC*mec* IV *S. epidermidis* strains exist in the dental plaque of healthy people and may be a potential risk for the transmission of antibiotic resistance.

## Introduction

Oral microflora, the ecological community of oral commensal, symbiotic, and pathogenic microorganisms, is the second largest microbial community in the human microbiome. High-throughput whole-genome sequencing and analysis have further revealed the complex diversity of the human oral microbiome, with up to 1 179 oral microbial taxa identified so far.^1^ These symbiotic and pathogenic microorganisms can reside in the oral cavity in planktonic (common in saliva) and biofilm form (common in dental plaque). Owing to indiscriminate or poor use of antibiotics throughout the world, antibiotic resistance, a pressing international public health problem, has been increasingly associated with increased morbidity, mortality and healthcare costs in recent years.^2,3^ The idea that oral microflora may serve as a latent reservoir of antibiotic resistance genes (ARGs) has been debated for some time. As shown in previous clinical data, multidrug-resistant bacteria have been found in oral microflora, and many of their ARGs have been found to be located on mobile genetic elements capable of broad horizontal gene transfer between different bacteria in bacterially diverse biofilms.^4,5^ In addition, there is a close connection between oral microbial pathogens and other systemic diseases, such as digestive system diseases, cardiovascular diseases, premature labor, mental illness, diabetes, and arthritis.^6^ Therefore, it is important to understand which genes the normal human oral microflora contains, and evaluate the possibilities for gene transfers to pathogenic microbes.

As a common human commensal microorganism, *Staphylococcus epidermidis* ubiquitously colonizes skin and wet mucosal surfaces, and has become a frequent and important opportunistic pathogen, particularly in immunocompromised patients.^7,8^ The presence of specific ARGs in this species, and its tendency to form biofilms, contribute to its pathogenicity and the complexity of treating its infections, which presents a significant burden for the public health system.^9,10^ Resistance to methicillin, a preferred antibiotic against staphylococcal infections, has been detected in 75%–90% of all hospital isolates of *S. epidermidis* carrying the phylococcal cassette chromosome *mec* (SCC*mec*) which contains the methicillin resistance gene (*mecA* gene), even higher than the corresponding rate for *Staphylococcus aureus* (40%–60%) in many countries, including China.^7,11^ In recent research, Multilocus Sequence Typing (MLST) has been applied to gain more information on the evolution, population structure, and long-term global epidemiology of *S. epidermidis*.^12^ To gain insight into the characteristics of its specific antibiotic resistance and biofilm formation, susceptibility testing, resistance gene and biofilm-associated virulence gene detection, have also been particularly important and necessary.^13,10^ Previous studies on its molecular epidemiology have shown that *S. epidermidis* strains show high levels of diversity, and most isolates belong to Clonal Complex 2 (CC2), which includes the most frequently isolated ST2 containing *IS256* insertion sequences, *ica* genes, and the capacity to form biofilms.^7^

A few studies have shown that oral *Staphylococci* or Methicillin Resistant *Staphylococci* (MRS) colonization can be isolated and identified in human oral microflora at different percentages.^14,15^ But detailed information on molecular epidemiology and susceptibility profiles is lacking. Therefore, one of the aims of this study is to isolate Methicillin-Resistant *S. epidermidis* (MRSE) strains from the dental plaque of a normal, healthy human population, and culture them in selective mediums. The focus will then be on the analysis of the molecular epidemiology, biofilm formation, resistance mechanisms, and susceptibility profiles of the MRSE strains, in order to provide the basic data to explore the potential transmission of resistance genes throughout the oral microflora.

## Results

### Characteristics of β-lactam-resistant strains isolated from dental plaque

The specimens were inoculated onto BHI blood agar and grown for 24 h at 37 °C in the presence of oxygen. A total of 226 β-lactam-resistant isolates, 37 species of bacteria and four species of fungus, were collected during the study period. The proportion of Gram-positive strains was 72.12% (163 isolates), higher than the 26.11% (59 isolates) Gram-negative strains and the 1.77% (4 isolates) of fungi (Fig. S1b). Of the 226 β-lactam-resistant isolates collected, *Staphylococcus epidermidis* (35 isolates), *Rothia mucilaginosa* (25 isolates), *Microbacterium sp* (20 isolates), *Stenotrophomonas maltophilia* (20 isolates), *Lactobacillus casei/reuteri* (15 isolates), *Corynebacterium argentoratense* (15 isolates), *Lactobacillus rhamnosus* (12 isolates), and *Chryseobacterium indologenes* (10 isolates) represented the major proportion. All other species had less than 10 resistant isolates each. The full characteristics of the β-lactam-resistant isolates are shown in Fig. S1a and Table S2. The detection rate of MRSE in the study is 8.01% (25/312). The abundance of MRSE detected from positive individuals is 2.8%–5.7% (1/35-2/35). In other words, one or two colonies were taken from each positive sample.

### Characteristics of antimicrobial resistance of MRSE

**Fig. 1:**
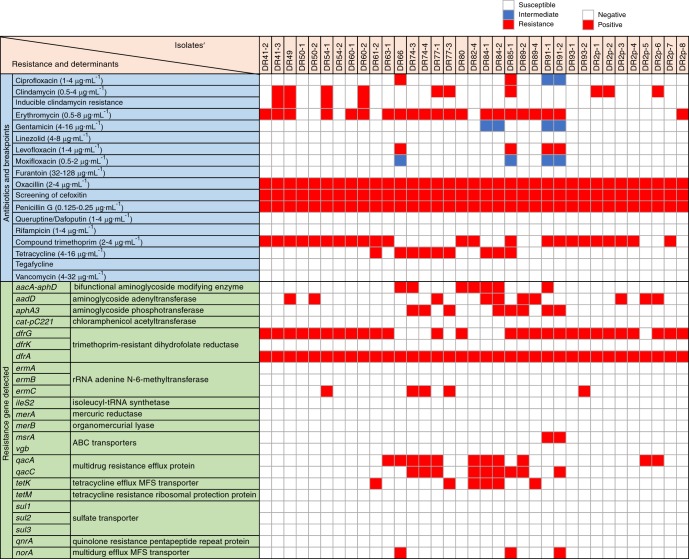
Upper part, distribution of common antibiotic resistances for the MRSE collection studied. The red, blue, and white blocks represent resistance, intermediate, and susceptible, respectively. Lower part, main antibiotic resistance related mutations and horizontally acquired resistance determinants of the 35 MRSE isolates. The red blocks denote that the detection of the gene is positive. ABC, ATP-binding cassette; MFS, major facilitator superfamily

The results of our antimicrobial susceptibility and associated resistance gene screening of the recovered MRSE strains are shown in Fig. 1. All 35 MRSE isolates were susceptible to some non-β-lactam antibiotics, such as linezoli, furantoin, queruptine/dafoputin, rifampicin, tegacycline, and vancomycin. All isolates were nonsusceptible to oxacillin and penicillin G. Most of them were also resistant to trimethoprim (23/35; 65.7%) and erythromycin (19/35; 54.3%), whereas nonsusceptibility rates were lower for clindamycin (10/35; 28.6%), tetracycline (9/35; 25.7%), and others. 60% (21/35) of the MRSE strains were multidrug-resistant *S. epidermidis* strains (resistant to three or more types of antibiotics simultaneously). In terms of Aminoglycoside (AM) resistance, the proportion of *aacA-aphD*, *aadD*, and *aphA3* gene-positive strains came to 20%, 28.6%, and 22.9%, respectively. Trimethoprim resistance was widespread, as the *dfrA* gene was positive in 100% of the isolates, and 80% of the isolates were also *dfrG* gene-positive. For erythromycin resistance, the *ermC gene* was present in 14.3% of the strains, and the *msrA* gene in 5.7%. As for resistance to tetracyclines and fluoroquinolones, the *tetK* gene was found in 17.1% of the isolates, and the *norA* gene in 8.6%. Finally, multidrug resistance efflux proteins encoded by the *qacA* and *qacC* genes were detected in 31.4% and 25.7% of the strains, respectively.

### Analysis of MLST sequencing profiles of oral MRSE

**Fig. 2:**
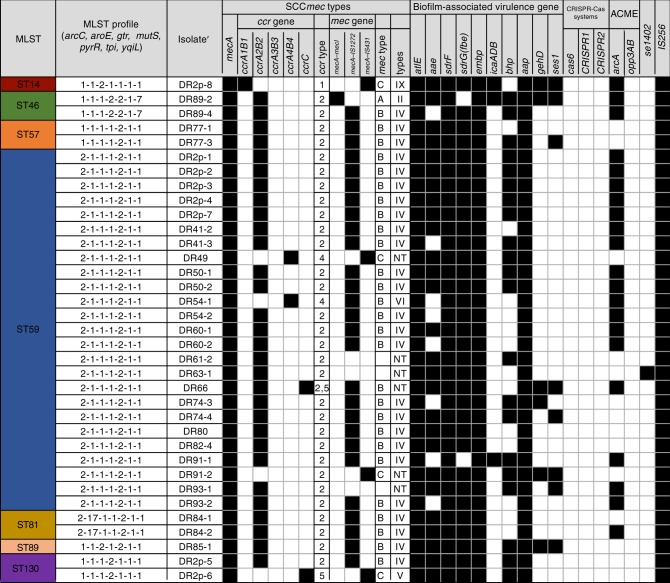
Detailed distribution of MLST genotypes, SCC*mec* types, biofilm-associated virulence genes, ACME allotypes and other potential virulence gene in the 35 MRSE isolates of this study. The black blocks denote that the detection of the gene is positive

**Fig. 3:**
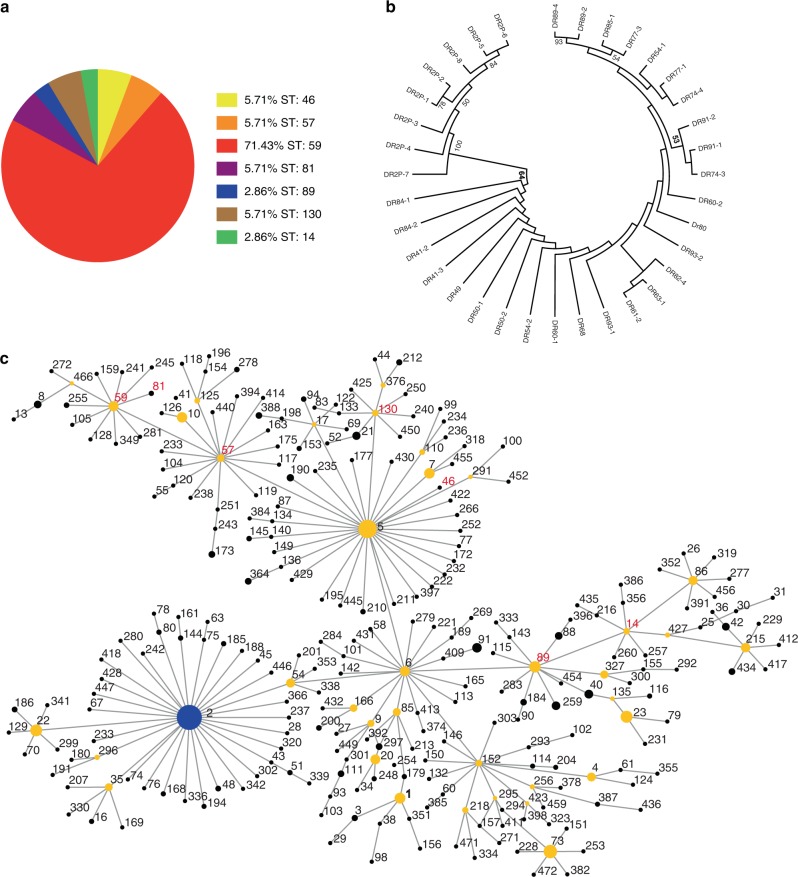
Characteristics of MLST genotypes for the collection of 35 oral MRSE isolates. **a** Prevalence of MLST genotypes in the studied 35 MRSE isolates. **b** Phylogenetic analysis of seven housekeeping genes. Strain names are preceded by the names of the bacterial species. The branch length indicates the distance. Numbers at the branching points represent the percent occurrence in 1 000 random bootstrap replications of neighbor-joining analyses. Values of less than 50% are not shown. **c** eBURST analysis of *S. epidermidis* CC2 using all STs available in the MLST database as of June 2019. Each ST is represented by a dot, and lines connect single-locus variants. The blue dot (ST2) represents the putative founder of CC2. The yellow dots represent putative subgroup founders. The red STs represent those recorded in this study

Thirty five isolates were genotyped by MLST. In total, seven different sequence types were identified, including ST14, ST46, ST57, ST59, ST81, ST89, and ST130 (Fig. 2). ST59 was the most common sequence type in dental plaque from healthy people in this study, comprising 71.43% (25/35) of the typed isolates, distantly followed by ST46 (2/35; 5.7%), ST57 (2/35; 5.7%), ST81 (2/35; 5.7%), ST130 (2/35; 5.7%), ST14 (1/35; 2.9%), and ST89 (1/35; 2.9%) (Fig. 3a). The eBURST algorithm clustered our STs into one major clonal complex (CC2-II), and a total of seven STs had been previously recorded in the MLST database (as of June 2019) (Fig. 3c). In our phylogenetic analysis, low bootstrap values showed that there was a relatively distant genetic relationship between most of the isolates (Fig. 3b). Cluster CC2-II can be further sub-divided into two groups, represented in this study by CC2-II-5 (5/7;71.4%) and CC2-II-89 (2/7; 28.6%).

### Detection of mecA and SCCmec genotype profiles

All 35 of the MRSE isolates were positive for the *mecA* gene. Among them, the SCC*mec* types were as follows: one (2.86%) was SCC*mec* type II, 25 (71.43%) were assigned to type IV, one (2.86%) was type V, one (2.86%) was type VI, one (2.86%) was type IX, and six isolates were non-typeable. In the six non-typeable isolates, we identified two isolates carrying SCC*mec* structures with new associations between the *ccr* complex and *mec* complex, that may correspond to two novel SCC*mec* structures: *mec* complex C associated with *ccrAB4* (one isolate), and *mec* complex B associated with *ccrC* (one isolate). The remaining four isolates carried SCC*mec* types that were non-typeable by the method used. (Fig. 2)

### Analysis of biofilm-forming ability and detection of biofilm-associated virulence genes, IS256, and ACME elements

**Fig. 4:**
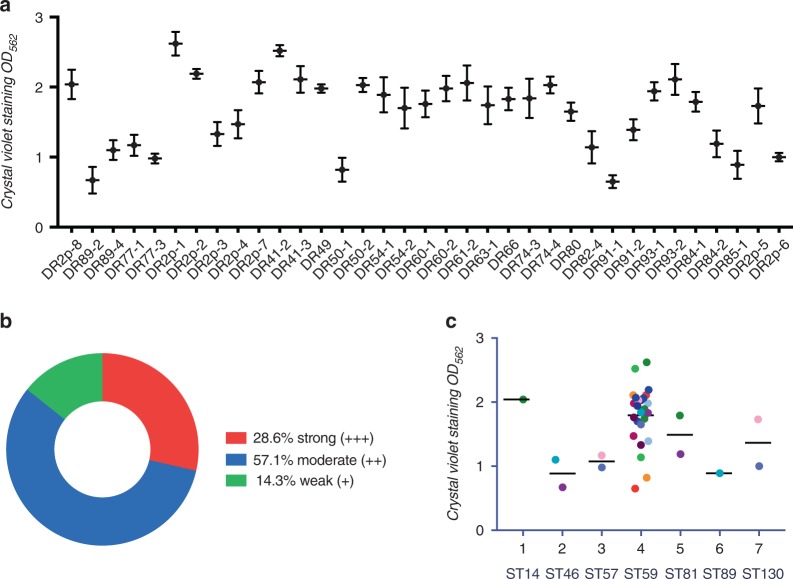
Biofilm phenotype for the collection of 35 oral MRSE isolates. **a** Measurement of 35 MRSE isolates’ biofilm biomasses by crystal violet staining. *S. epidermidis* strains were cultured in sterile 96-well microtiter plates and absorbance was recorded at 562 nm with microplate reader (Gene, Hong Kong, China). **b** Prevalence of biofilm-forming ability of the 35 MRSE isolates in this study. **c** Measurement of seven STs’ biofilm biomasses by crystal violet staining. *S. epidermidis* strains were cultured in sterile 96-well microtiter plates and the absorbance was recorded at 562 nm with microplate reader (Gene, Hong Kong, China)

**Fig. 5:**
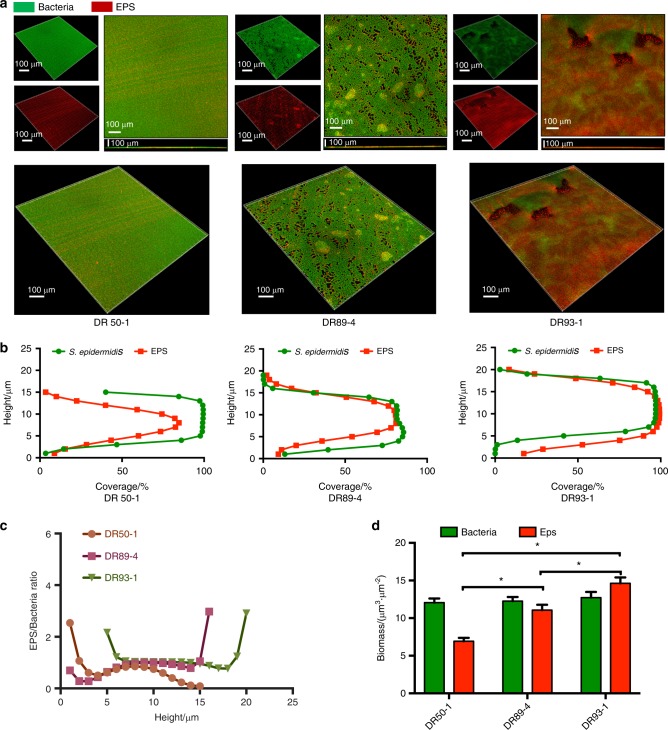
Biofilm architecture and EPS distribution of S. epidermidis strains observed by confocal microscopy. **a** Double-labeling of 24 h *S. epidermidis* biofilms. Green, bacteria (SYTO 9); red, EPS (Alexa Fluor 647). The three-dimensional reconstruction of the biofilms and the quantification of bacteria/EPS biomass were both performed with IMARIS 7.0.0. **b** The distributions of EPS and bacteria at different heights. **c** Quantification of bacteria/EPS biomass was performed with IMARIS 7.0.0. Results are the average of five randomly selected positions of each sample and are presented as mean ± standard deviation. *P* < 0.05. **d** The ratio of EPS to bacteria at different heights was quantified with IMARIS 7.0.0. Results are the average of five randomly selected positions of each sample and are presented as mean ± standard deviation. EPS, Extracellular Polymeric Substances

The ability to form biofilm was studied for 35 methicillin-resistant *S. epidermidis* strains. 28.6% showed strong biofilm-forming abilities (+++), 57.1% were moderate (++), and 14.3% were weak (+) (Fig. 4a, b). ST59 *S. epidermidis* isolates generally possessed a strong ability to form biofilms (Fig. 4c). We further examined the three representative strains with different biofilm-forming abilities using SEM and confocal laser scanning microscopy (Fig. 5a–d, S2). All the methicillin-resistant *S. epidermidis* isolates carried not less than half of biofilm-associated genes in this study, and 88.66% (31/35) harbored six or more of these genes, but, interestingly, none had all ten biofilm-associated genes. The genes *aap*, *atlE*, *embp*, and *sdrF* were detected in all 35 isolates. The *bhp*, *aae*, and *fbe* genes were frequently found, in percentages ranging from 60% to 94.3%. However, the *icaADB*, *gehD*, and *sesI* genes were less frequently found, ranging in percentages from 8.6% to 22.9%. In brief, 11 different genetic profiles of the whole biofilm-associated genes were detected in this study. The majority of the profiles (31.4%) was *gehD* (-), *sesI* (-), *icaADB* (-), *aae* (+), *aap* (+), *atlE* (+), *bhp* (+), *embp* (+), *sdrF* (+) and *fbe* (+). All of the isolates (100%) were found to be positive for *IS256*. Among these isolates, 21 of them harbored the *arcA* gene only (ACME II), and none of them harbored the *opp3AB* gene (Fig. 2).

## Discussion

With the widespread prescription of antibiotics today, the noteworthy clinical and economic burden of antibiotic resistance has emerged and spread worldwide. Antibiotic-resistant bacteria have been increasingly isolated from odontogenic infections in recent years,^16,17^ and have affected the microbiome in the human oral cavity, and elsewhere as well. Although dental plaque biofilms are considered to be a repository of drug-resistant bacteria and drug-resistant genes, we are still not yet certain about the prevalence, genotypes, and unique characteristics of drug-resistant genes in the mouth. More attention should be paid to oral antibiotic-resistant bacteria.^18,19^ In this study, we collected a total of 226 β-lactam-resistant isolates: 37 species of bacteria and four species of fungus. 35 MRSE isolates, the majority of the β-lactam-resistant isolates, were further researched in terms of molecular typing, drug resistance, biofilm phenotype and genotype and so on.

The prevalence of MRSE strains in this study was particularly high, and we speculated that there were likely two major factors responsible for this high recovery. Firstly, MRSE bacteria are simply naturally resistant to β-lactam antibiotics. Methicillin is a β-lactam antibiotic which impairs the development of bacterial cell walls by interfering with transpeptidase enzymes, or the Penicillin-Binding Proteins (PBPs). In MRSE bacteria, the Staphylococcal Cassette Chromosome *mec* (SCC*mec*), containing the *mecA* gene, encoded a PBP, PBP2a, which possessed lower affinity for methicillin in contrast with other PBPs.^20,7^ Secondly, *S. epidermidis* is one of the dominant symbiotic bacteria found in the human nasal cavity,^9^ and, as another entrance to the respiratory tract, microbial pathogens in oral cavity and nasopharynx were closely related.^5^ Research has shown that methicillin-resistant *Staphylococci* have been detected in both the oral cavities and the nasopharynxes of children with respiratory diseases.^21^ Therefore, we hypothesize that biofilm-positive *S. epidermidis* exists in dental plaque biofilms as well as in the nasal passages.

Most of the MRSE isolates in this study were multidrug-resistant *Staphylococcus epidermidis* strains, and their large amounts of acquired plasmid-borne genes and sequence variations conferred their resistances to multiple antibiotic types. The *aacA-aphD*, *aadD*, and *aphA3* genes were all associated with chemical modifications to Aminoglycoside-Modifying Enzymes (AMEs), leading to Aminoglycoside (AM) resistance, in this study. AME genes were frequently transferred to mobile elements for instance plasmids and integrons, together with other resistance genes.^22^ Plasmid-borne transposons with AME genes also contained the *Tn4031* gene in MRSEs,^23^ *Tn 4001* in MRSAs, and *Tn 5281* in *Enterococcus faecalis* strains.^24,25^ The presence of the *dfrG* and *dfrA* genes, which encode variant DHFRs located on exchangeable genetic elements, were found to be responsible for trimethoprim resistance in this study. A 100% detection rate in MRSE isolates in this study was found to be in accordance with the notion that the *dfrA* gene is currently deemed to be one of the decisive factor for trimethoprim resistance in *Staphylococcus* species isolated from humans.^26^ The *dfrG* gene was reported in an MRSA clone in a Thailand hospital in 2005, and an outbreak of MRSA exhibiting *dfrG* occurred shortly afterward in Africa and Europe.^27,28^ The *msrA* gene (encoding ATP-dependent transporters), the plasmid-borne *ermC* gene (for erythromycin methylase), the *tetK* gene (tetracycline efflux MFS transporter) and the *norA* gene (multidrug efflux MFS transporter) all contributed to erythromycin resistance and intermediate resistance to gentamicin, tetracyclines, and fluoroquinolones, respectively.

The antibiotic susceptibility test (AST) results as well as the detection of a series of AMR genes on these MRSE have been correlated to further give us new insights into MRSE drug resistance. However, as it is well known that the prediction of antibiotic resistance phenotypes based on genotypes is not always accurate, and vice versa. Some marker AMR genes can be detected, but its corresponding antibiotic resistance phenotype cannot be detected. For this limitation, the AST method could be improved next-time. Since MIC is based on growth while MIC-MA is based on metabolic activity,^29^ the combination of these two parameters promise to detect the non-growth but metabolically active (NGMA) category of bacteria-drug interactions, and to provide a very novel, and more comprehensive and in-depth, understanding of how the MRSE strains are resistant to these drugs. At the same time, we can further focus on a particular class of antibiotic-resistant genes to examine in single-base resolution the sequence heterogeneity of these genes.^30^ In a word, we could combine a sequence-level, higher-resolution view of ARGs with the improved AST method, combination of the MIC and MIC-MA parameters, to derive a comprehensive picture of drug resistance.

Moreover, we have explored the existences of CRISPR-Cas systems in our isolates, which can not only protect bacteria from phage infection, but may also help bacteria resist the horizontal transfer of antibiotic-resistant genes, leading to a diminished drug-resistance or virulence of *Staphylococci*.^31,32^ These adaptive immunity systems which act against invading genetic elements such as viruses and plasmids were not found in any of our isolates. As a result, more invading genetic elements can invade these bacteria more easily and thus its genetic diversity was increased. This absence of CRISPR-Cas systems in our MRSEs confirmed their enormous diversity, and the abundance of horizontally acquired resistance genes in these bacteria. More importantly, these horizontally acquired resistance genes could also be delivered to other pathogenic bacteria when appropriate.

A potential need for *S. epidermidis* bacteria to adapt to challenges from different environments may spur their genetic diversity, resulting in increased frequency of horizontal gene transfer and transmission of mobile gene elements.^10^ The degree of genetic diversity in this study was determined by the strain inclusion criteria for application, and the range of geographic origins of the samples. A higher level of genetic diversity was surveyed in 35 *Staphylococcus epidermidis* strains. The ratio of STs studied in this research (seven STs among 35 strains) was slightly higher than has been formerly published results (8 STs among 40 linezolid-resistant Coagulase-Negative Staphylococcus strains) from a single clinical origin,^13^ and lower than that of a cohort of samples gathered from several countries in the world, in a larger study. (74 STs among 217 strains).^10^

The sequence type ST59 (CC2-II-5) (71.43%) predominated in this investigation, in contrast to the sequence type ST2 (CC2-I) which is generally the most extensive distributed type in the infected sample, owing to the theoretical basis that the whole ST2 (CC2-I) strains carry *ica* genes and *IS256* insertion sequences,^10,11^ correlating highly with invasiveness.^33–37^ These differences may possibly have been caused by the diversity of our specimen selection criteria. Dental plaque specimens in this study were collected from a healthy population without any obvious clinical symptoms of *S. epidermidis* infection. In other studies, traces of the ST59 (CC2-II-5) genotype were also found in infected specimens, but its proportions were significantly lower, at 3.7% (*n* = 3), 6.7% (*n* = 2), and 7% (*n* = 5).^13,20,10^ Interestingly, *IS256* insertion sequences were positive in all of the MRSE isolates in this study. Containing *IS256* insertion sequences was relevant to biofilm formation and multidrug resistance, thus providing advantages in rapid adaptation and flexibility to the bacteria, in terms of responding to changing environmental conditions.^34,35^

A single genetic lineage (CC2), containing a large number of STs, occupied the majority of the population analyzed in this study. This phenomenon could be the result of high rates of recombination.^10^ By means of long-term recombinational replacements in the general gene pool, abundant alleles became a shared resource and the homogenization of the population arises in subgroup founders. Furthermore, in the minimum evolution tree the deficiency of phylogenetic consistency also approved the above analysis results (Fig. 3b). Furthermore, the broad habitat range of *S. epidermidis* provides more constant contact and exchange with other bacteria, contributing to its high levels of SCC*mec* acquisition and homologous recombination.

Concerning the SCC*mec* types found in this study, SCC*mec* type IV was the most often obtained by *S. epidermidis* strains (25 out of 35 isolates), in concert with the SCC*mec* type already observed in MRSAs and MRSEs with enhanced mobilities.^10^ Moreover, the proportion of SCC*mec* type IV increased significantly from 12.4% (68 isolates) to 45.3% (185 isolates) between 2010 and 2014 in Japan.^38^ SCC*mec* type IV (21–24 kb), merely possessed the *mecA* gene in general, and was considered to be movable and involving the site-specific recombinase genes.^10^ Its mobility and small size are partially responsible for its common occurrence. We have found that identical or affinitive STs have been discovered to contain different SCC*mec* types (II, V, VI, NT), and the identical SCC*mec* type was also discovered within various, often irrelevant STs. MLST is an excellent tool that has been widely used for estimating the transference of SCC*mec*.^10,39^ The combination of SCC*mec* types and the chromosomal backgrounds have been applied to define MRSA or MRSE clones.^39^ ST59-SCC*mec* IV isolates, abbreviated as ST59-IV, were the majority of the population analyzed in dental plaque from a healthy population, in this study.

Another factor favoring horizontal gene transfer is biofilm formation via cell–cell interactions. Biofilms, the major virulence factor of *S. epidermidis*, are pluricellular, surface-attached clusters of microorganisms. In this study, we detected biofilm-associated virulence genes at different stages of biofilm-formation, including initial attachment to abiotic surfaces or to matrix proteins (*atlE*, *aae, bhp* or *sdrF*, *fbe*, *ebp*, *atlE*, and *aae*), and intercellular aggregation (*icaADB*, *bhp*, *aap*).^7^ The characteristics of biofilm-associated virulence genes in MRSEs detected in dental plaque were analyzed. Firstly, the presence of the *ica* locus was detected in only a few of the isolates (3/35). Secondly, all of the isolates in the present study were *atlE*, *sdrF*, *ebp*, and *aap* gene-positive, and only two of the isolates were *sdrG* (*fbe*) gene-negative. What is more, the *aae* and *bhp* genes were detected in most of them. Therefore, we found that MRSEs isolated from dental plaque in this study possessed a strong capacity for initial adhesion. The positive detection of *atlE* and *bhp* genes determines the hydrophobicities of bacterial cell surfaces, helping them attach to abiotic surfaces such as composite resin and titanium implants.

MRSE isolates lacking the crucial *ica* gene for biofilm formation were still detected in oral biofilms, for which we propose two possible reasons. One is that the *ica*-negative MRSE isolates mostly participated in early colonization. Another is that *ica*-negative strains can also form biofilms, which has been confirmed elsewhere,^40^ entirely or additionally mediated by particular surface proteins Bhp and Aap (positive in all of the strains).^41,42^ In addition, 21 (60%) of the 35 isolates in this study harbored the genetic island ACME II, a similar high prevalence to the findings in another study.^43^ The *Staphylococci* species obtaining of the ACME mobile element seems to possess an advantages in colonization, but not in pathogenicity.^44^

*Staphylococcus epidermidis* ubiquitously and naturally colonizes skin. Unlike MRSE from dental plaque, *Staphylococcus epidermidis* strains from skin own a higher degree of distributed diversity (74 STs) and mostly belong to the most frequently isolated ST2 due to containing *IS256* and *ica* genes.^7^ The epithelial MRSE strains also have obtained several other antibiotics resistance, containing erythromycin, gentamycin, chloramphenicol, rifamycin, fluoroquinolones and tetracycline.^45^ Most ARGs are plasmid-encoded.^46^ In general, MRSE from dental plaque and from skin have many similarities that they are biofilm-positive multiple-antibiotic-resistant CC2-SCC*mec* IV *S. epidermidis* strains, but MRSE strains from skin own a higher positive rate of *ica* gene and higher level of STs diversity and the most frequently isolated STs is ST2, the putative founder of CC2.

In conclusion, our research has confirmed that there were multifarious β-lactam -resistant bacterial strains found in human dental plaque from members of a healthy population, mainly including biofilm-positive multiple-antibiotic-resistant ST59-SCC*mec* IV *S. epidermidis* strains. These strains’ strong capabilities to adapt to changing oral environments and abundant mobile genetic elements make them a potential pathogenic risk. Our investigation into genomics and susceptibility profiles of these Methicillin-resistant *S. epidermidis* isolates will hopefully prove beneficial in providing a new perspective on hospital infection control.

## Materials and methods

### Specimen collection

This was a forward-looking study relating to the dental plaque from healthy volunteers who had provided informed consent. The inclusion criteria were as follows: Age of 20–30 years; Gender unlimited; No food intake within the preceding two hours; No history of drug use. If there is any recent medication or vitamin supplement intake, the name of the active ingredient and the dose and frequency of intake must be indicated at the next stage; No history of smoking or drinking. Anyone receiving tobacco or alcohol must report the type and quantity; No Dental caries Missing and Filling Surface (DMFS); No cancer, diabetes, chronic respiratory diseases, and cardiovascular and cerebrovascular diseases including hypertension, stroke and coronary heart disease; Normal overall physical and mental condition.

The exclusion criteria were as follows: Used systemic antibiotics, cortisone hormones (intramuscular, oral, nasal spray or inhalation), cytokines that stimulate the body’s immune system such as interleukinin, immunosuppressants such as methotrexate, or large doses of probiotics (>10^8^ CFU per day) in the past six months; Used topical antibiotics within 7 days; HIV, HBV and HCV positive; Pregnant and nursing women; Calculus index (CI) ≥ 2; A fungal infection of the mouth.

Dental plaque specimens were obtained from tooth cervical sites using a normal aseptic scraper or Moore00 aseptic scraper, which were stored in 200 μL Tris-EDTA (Ethylenediaminetetraacetic acid) buffer (TE) buffer temporarily, then used to directly inoculate BHI blood agar 24 h with 4 μg·mL^−1^ Meropenem (Mem) at 37 °C, in the presence of oxygen.

### MRSE strains collection and identification

Each colony that formed on the BHI blood agars with 4 μg·mL^−1^ Mem was picked and transferred into liquid BHI medium with 4 μg·mL^−1^ Meropenem (Mem) at 37 °C, to purify it based on its colony morphology. Bacterial colony 16S rDNA sequencing was applied to identify the bacteria;^47^ the primers used for this procedure are shown in Supplemental Table 1. 35 strains of *Staphylococcus epidermidis* were selected and identified by using the Vitek-2 system (bioMérieux, Marcy l’Etoile, France).

### MRSE susceptibility testing and resistance gene detection

Minimal inhibitory concentrations (MICs), or breakpoints, of 18 antibiotics and inducible tests (Ciprofloxacin, Clindamycin, Inducible clindamycin resistance, Erythromycin, Gentamicin, Linezolid, Levofloxacin, Moxifloxacin, Furantoin, Oxacillin, Screening of cefoxitin, Penicillin G, Queruptine or Dafoputin, Rifampicin, Compound trimethoprim, Tetracycline, Tegafycline, Vancomycin) were determined for each MRSE isolate, in the light of the Clinical and Laboratory Standards Institute (CLSI, Wayne, MI, USA) guidelines (M100-S25).^48^ Following this, 35 *Staphylococcus epidermidis* strains were analyzed for the existence of ARGs *ermA*, *ermB*, *ermC*, *sul1*, *sul2*, *sul3*, *dfrG*, *dfrK*, *dfrA*, *tetK*, *tetM*, *aacA-aphD*, *aadD*, *aphA3*, *merA*, *merB*, *msrA*, *vgb*, *qacA*, *qacC, qnrA*, and *norA* for all isolates nonsusceptible to erythromycin, compound trimethoprim, tetracycline, aminoglycosides, fluoroquinolone, mercury, and others.

### Multilocus sequence typing

Genomic DNA for Polymerase Chain Reaction was collected by lysis buffer at 37 °C for 1.5 h, and then at 95 °C for 15 min. MLST was implemented according to the novel MLST scheme described,^12^ based on the sequencing of internal fragments of seven housekeeping genes, including *arcC*, *aroE*, *glpK*, *gmk*, *pta*, *tpiA*, and *yqi*. The fragments were amplified by PCR using primers for highly conserved regions. The primers for these seven housekeeping genes are shown in Supplemental Table 1. According to the *S. epidermidis* MLST database (http://sepidermidis.mlst.net/) the numbers of alleles and sequence types (STs) were assigned. STs were classified in the same group only if they shared identical alleles at six or more of the seven MLST loci. Evolutionary analyses were conducted by MEGA7 and the eBURST method (http://eburst.mlst.net/, Edward, UK).^49^ In MEGA7, molecular phylogenetic analysis was deduced using the Maximum Likelihood method, based on the Kimura 2-parameter model. With the eBURST algorithm, all members of the group were considered to have descended from the same founding genotype. The bootstrap consensus tree and the statistical confidences for the founders all were assessed using 1 000 bootstrap resamplings.

### The mecA gene detection and SCCmec typing

According to procedures detailed, the carrying situation of the *mecA* gene and SCC*mec* typing were implemented.^50,51^ The combination of the types of *ccr* (encoding for recombinases) and *mec* (encoding for beta-lactam resistance) class allowed the identification of the type of SCC*mec* (I to IX). While no PCR amplification occurred for any of the primer pairs used, the *mec* complex was non-typeable. When a positive PCR amplification signal was obtained merely for βc/αc primers, or no PCR amplification occurred for any of the primer pairs used, the *ccr* complex was non-typeable. If the *ccr* complex, the *mec* complex, or both, were non-typeable, the SCC*mec* type was non-typeable.^50–52^ *S. epidermidis* RP62A and ATCC12228 were served as controls.

### PCR assays for biofilm-associated genes, IS256 and ACME elements

The carrying situation of biofilm-associated genes: *aap*, *atlE*, *aae*, *fbe*, *gehD*, *bhp*, *embp*, *sdrF*, *sesI*, *icaADB*, *IS256*, and ACME (Arginine Catabolic Mobile Element) allotypes was done by PCR. All isolates were tested for the presence of the *arc* and *opp-3* genes, to assess the presence of the ACME. ACME allotypes were classified as: ACME-I, containing both the *arc* and the *opp-3* gene clusters; ACME-II, containing *arc* but not *opp-3*; and ACME-III, containing *opp-3* without *arc*.^44,53^ *S. epidermidis* RP62A and ATCC12228 served as controls. All PCR fragments were visualized by agarose gel electrophoresis and GoldView™ staining.

### Assessment of biofilm biomass by crystal violet staining

Assessment of biofilm biomass by crystal violet staining were performed as previously described.^54^ An overnight culture of *S. epidermidis* was diluted at 1:20 in BHI broth (Difco, Sparks, MD, USA) with 0.5% glucose. the diluted bacterial suspensions were inoculated into sterile 96-well microtiter plates for 24 h at 37 °C. After incubation, the planktonic bacteria were removed, and 96-well microtiter plates were washed by 0.9% Phosphate Buffered Saline (PBS) for three times. Then, the biofilm was fixed with 10% formaldehyde and washed again. 200 μL 0.1% (g·L^−1^) crystal violet was added to stain the biofilm, and it was incubated for 10 min with gentle rocking. The wells were rinsed twice with 0.9% PBS, and 200 μL 30% acetic acid was added to the wells to solubilize the dye under gentle rocking for 15 min. Finally, the acetic acid was transferred to a new plate and the absorbance at 562 nm with microplate reader (Gene, Hong Kong, China) was recorded. The comparative analyses were performed based upon the optical density (OD) of bacterial films according to Stepanović et al.^55^ All isolates were classified into the following categories: strong (+++), moderate (++), weak (+), and nonproducer of biofilm (−) based upon the ODs of bacterial films. We defined the cut-off OD (OD_C_) for the microtiter-plate test as three standard deviations above the mean OD of the negative control (ATCC12228). The OD_C_ of this study approximately equal to 0.5. Strains were classified as follows:

OD ≤ OD_C_ = 0.5 biofilm (−)

OD_C_ = 0.5 < OD ≤ 2X OD_C_ = 1 biofilm weak (+)

2X OD_C_ = 1 < OD ≤ 4X OD_C_ = 2 biofilm moderate (++)

4X OD_C_ = 2 < OD biofilm strong (+++)

All tests were carried out three times and the results were averaged. *S. epidermidis* RP62A and ATCC12228 served as controls.

### Biofilm analysis and structural imaging

The bacterial cells were labeled with SYTO 9 (Molecular Probes, Invitrogen, Carlsbad, CA, USA) and EPS from *S. epidermidis* biofilms incubated for 24 h was labeled with Alexa Fluor 647 dye (Molecular Probes), as previously described.^56^ Biofilm images were captured with an Olympus FV 3000 confocal laser scanning microscope (Olympus, Tokyo, Japan) equipped with a 60 × oil immersion objective lens. Image collection gates were set to 495–515 nm for SYTO 9 and 655–690 nm for Alexa Fluor 647. Each biofilm sample was scanned at five randomly selected positions, and a confocal image series was generated by optical sectioning at each position. Three-dimensional reconstruction of the biofilms and the quantification of EPS/bacteria biomass were performed with IMARIS 7.0.0 (Bitplane, Zürich, Switzerland) and COMSTAT image-processing software^57^ was used for the analysis of the confocal image stacks and biomass quantification.

The architecture of *S. epidermidis* biofilms was also examined by SEM. The subcultured overnight culture of *S. epidermidis* biofilms were diluted as described above and inoculated onto glass coverslips placed in a 24-well cell culture plate for 24 h. Biofilms were fixed with 2.5% (wt/v) glutaraldehyde solution at 4 °C for 12 h, then serially dehydrated in ethanol and followed by sputter-coating with gold. Specimens were examined at ×1 000, ×5 000 and ×20 000 magnification.

### Statistical analysis

The above experiments were repeated for three times, and three groups of parallel controls were set for each repeated experiment. SPSS 20.0 software was used for statistical analysis, and one-way analysis of variance (ANOVA) was used to test and compare the differences between groups. If *P* < 0.05, the data in this group were statistically significant.

## Supplementary information

Figure S1

Figure S2

Table S1

Table S2
