# Supplementary figures and images for: Characteristics of oral methicillin-resistant Staphylococcus epidermidis isolated from dental plaque
Source: Int J Oral Sci. 2020 May 9;12:15. doi: 10.1038/s41368-020-0079-5 (PMC7210960; doi:10.1038/s41368-020-0079-5)

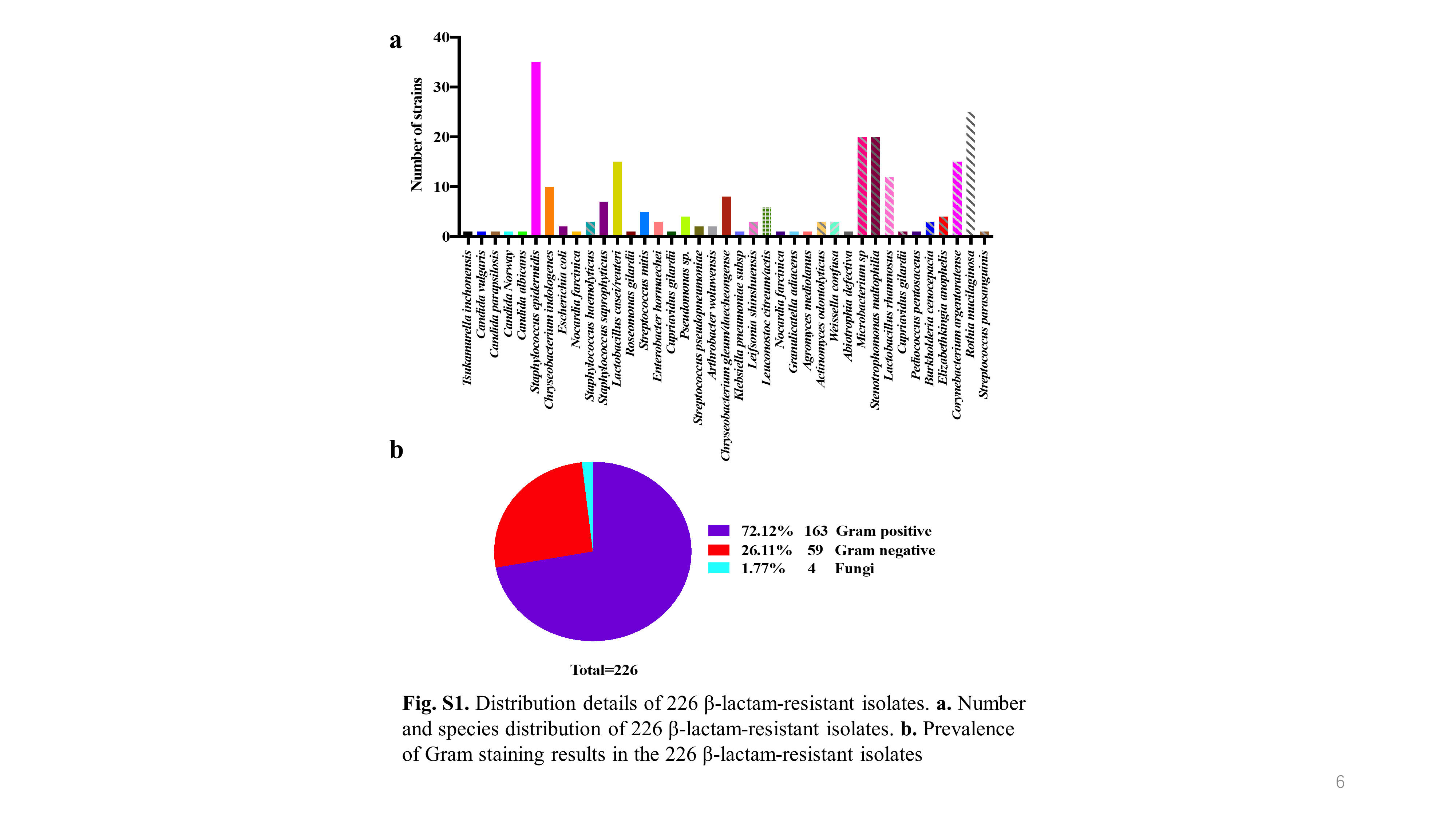

Supplement: Supplementary file 1 — Figure S1 [file 41368_2020_79_MOESM1_ESM.tif]

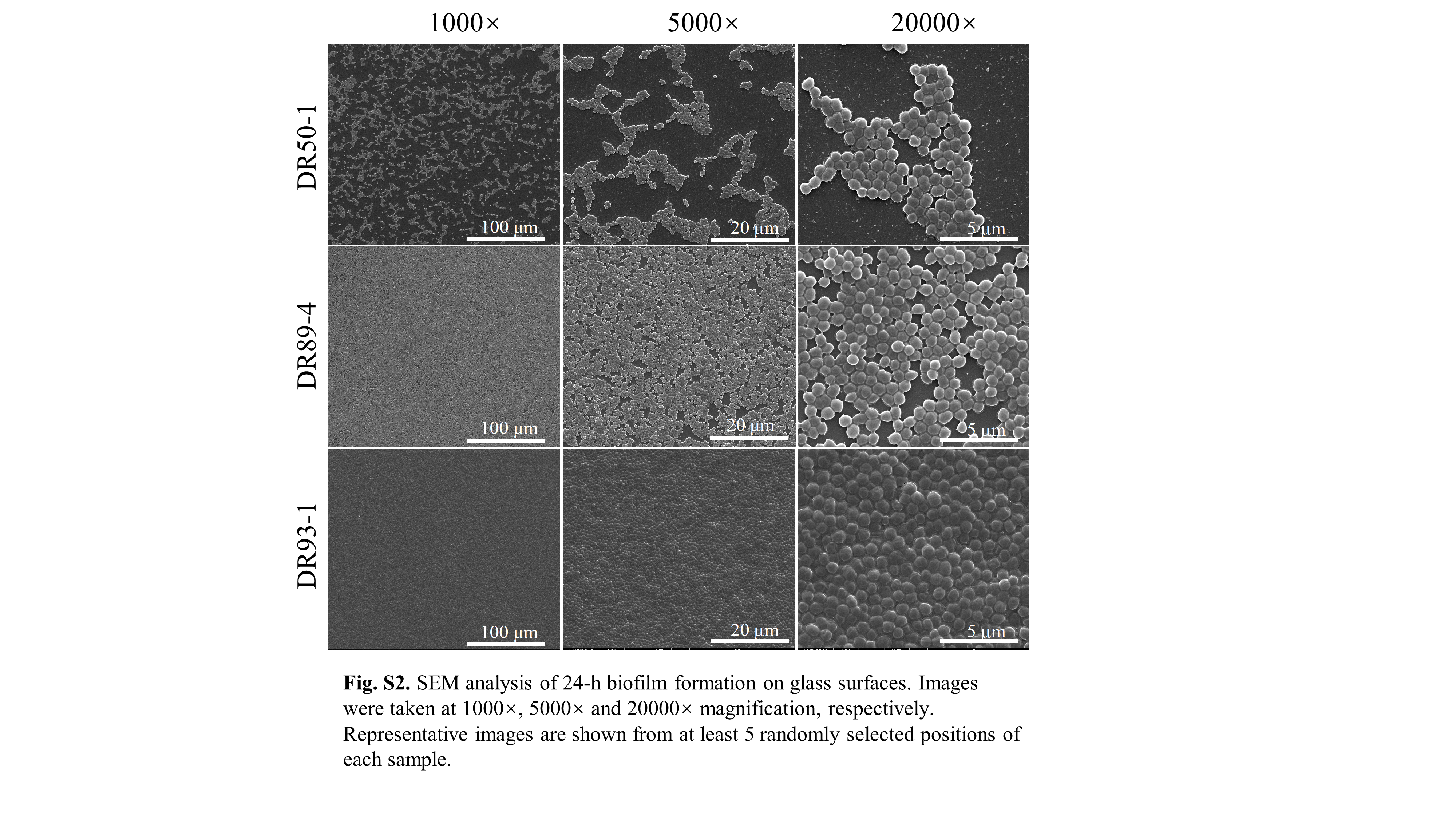

Supplement: Supplementary file 2 — Figure S2 [file 41368_2020_79_MOESM2_ESM.tif]
