# Supplementary material for: Characteristics of oral methicillin-resistant Staphylococcus epidermidis isolated from dental plaque
Source: Int J Oral Sci. 2020 May 9;12:15. doi: 10.1038/s41368-020-0079-5 (PMC7210960; doi:10.1038/s41368-020-0079-5)
Supplement: Supplementary file 4 — Table S2 [file 41368_2020_79_MOESM4_ESM.pdf]

Table S2. Detailed list of β-lactam antibiotic resistant bacteria in dental plaque biofilm

Isolate reference	Sampling Date	Strain Name	Gram Stain	BLAST Max	BLAST Query cover	Ident	Source	Incubation condition	Batch
DR41-1	20171202	*Abiotrophia defectiva*	Pos	1354	96%	95%	Human dental plaque	Aerobic	1
DR39-1	20171202	*Actinomyces odontolyticus*	Pos	2495	100%	99%	Human dental plaque	Aerobic	1
DR40-2	20171202	*Actinomyces odontolyticus*	Pos	1724	99%	99%	Human dental plaque	Aerobic	1
DR51	20171202	*Actinomyces odontolyticus*	Pos	2484	100%	99%	Human dental plaque	Aerobic	1
DR106-3	20171218	*Agromyces mediolanus*	Neg	2505	100%	99%	Human dental plaque	Aerobic	1
DR59-1	20171210	*Arthrobacter woluwensis*	Pos	2571	99%	99%	Human dental plaque	Aerobic	1
DR59-2	20171210	*Arthrobacter woluwensis*	Pos	2579	99%	99%	Human dental plaque	Aerobic	1
DR-2	20171101	*Burkholderia cenocepacia*	Neg	1720	98%	99%	Human dental plaque	Aerobic	1
DR-7	20171101	*Burkholderia cenocepacia*	Neg	1722	99%	99%	Human dental plaque	Aerobic	1
DR-8	20171101	*Burkholderia cepacia*	Neg	2468	100%	99%	Human dental plaque	Aerobic	1
DR89-1	20171213	*Candida albicans*	N	1733	98%	100%	Human dental plaque	Aerobic	1
DR87-2	20171213	*Candida inconspicua*	N	2507	100%	100%	Human dental plaque	Aerobic	1
DR-18	20171115	*Candida norvegensis*	N	1724	98%	99%	Human dental plaque	Aerobic	1
DR-17	20171115	*Candida parapsilosis*	N	1714/	100%	99%	Human dental plaque	Aerobic	1
DR-16	20171115	*Candida parapsilosis*	Neg		99%	99%	Human dental plaque	Aerobic	1
DR53-1	20171202	*Chryseobacterium daecheongense*	Neg	2449	99%	99%	Human dental plaque	Aerobic	1
DR56-1	20171202	*Chryseobacterium daecheongense*	Neg	2557	100%	100%	Human dental plaque	Aerobic	1
DR56-2	20171202	*Chryseobacterium daecheongense*	Neg	2534	99%	99%	Human dental plaque	Aerobic	1
DR38-1	20171202	*Chryseobacterium gleum*	Neg	2486	100%	99%	Human dental plaque	Aerobic	1
DR67-1	20171210	*Chryseobacterium gleum*	Neg	1219	93%	93%	Human dental plaque	Aerobic	1
DR67-3	20171210	*Chryseobacterium gleum*	Neg	835	98%	84%	Human dental plaque	Aerobic	1
DR79-2	20171210	*Chryseobacterium gleum*	Neg	2473	100%	99%	Human dental plaque	Aerobic	1
DR79-3	20171210	*Chryseobacterium gleum*	Neg	2492	100%	99%	Human dental plaque	Aerobic	1
DR-30	20171115	*Chryseobacterium indologenes*	Neg	1722	98%	100%	Human dental plaque	Aerobic	1
DR-31	20171115	*Chryseobacterium indologenes*	Neg	1722	98%	100%	Human dental plaque	Aerobic	1
DR95-1	20171213	*Chryseobacterium indologenes*	Neg	2479	100%	100%	Human dental plaque	Aerobic	1
DR95-3	20171213	*Chryseobacterium indologenes*	Neg	1400	97%	97%	Human dental plaque	Aerobic	1
DR98-1	20171218	*Chryseobacterium indologenes*	Neg	2479	100%	100%	Human dental plaque	Aerobic	1
DR103	20171218	*Chryseobacterium indologenes*	Neg	1736	99%	99%	Human dental plaque	Aerobic	1
DR107-1	20171218	*Chryseobacterium indologenes*	Neg	1735	99%	99%	Human dental plaque	Aerobic	1
DR107-2	20171218	*Chryseobacterium indologenes*	Neg	1722	99%	99%	Human dental plaque	Aerobic	1
44-1	20181210	*Chryseobacterium indologenes*	Neg	1722	99	99	Human dental plaque	Aerobic	2
DR-24	20171115	*Chryseobacterium indologenes*	Neg	2356	95%	90%	Human dental plaque	Aerobic	1
DR45-1	20171202	*Corynebacterium argentoratense*	Pos	2464	100%	99%	Human dental plaque	Aerobic	1
DR45-2	20171202	*Corynebacterium argentoratense*	Pos	2492	99%	99%	Human dental plaque	Aerobic	1
DR46-1	20171202	*Corynebacterium argentoratense*	Pos	1727	99%	99%	Human dental plaque	Aerobic	1
DR69-1	20171210	*Corynebacterium argentoratense*	Pos	2481	99%	99%	Human dental plaque	Aerobic	1
DR69-2	20171210	*Corynebacterium argentoratense*	Pos	2479	100%	99%	Human dental plaque	Aerobic	1
DR70-1	20171210	*Corynebacterium argentoratense*	Pos	2457	100%	99%	Human dental plaque	Aerobic	1
DR70-2	20171210	*Corynebacterium argentoratense*	Pos	2444	100%	99%	Human dental plaque	Aerobic	1
DR78-1	20171210	*Corynebacterium argentoratense*	Pos	2470	99%	99%	Human dental plaque	Aerobic	1
DR78-2	20171210	*Corynebacterium argentoratense*	Pos	2470	99%	99%	Human dental plaque	Aerobic	1
51-2	20181210	*Corynebacterium argentoratense*	Pos	1653	100	98	Human dental plaque	Aerobic	2
1-2	20181210	*Corynebacterium argentoratense*	Pos	1670	98	99	Human dental plaque	Aerobic	2
1-3	20181210	*Corynebacterium argentoratense*	Pos	1642	99	98	Human dental plaque	Aerobic	2
51-1	20181210	*Corynebacterium argentoratense*	Pos	1688	98	99	Human dental plaque	Aerobic	2
58-1	20181210	*Corynebacterium argentoratense*	Pos	1683	99	99	Human dental plaque	Aerobic	2
58-2	20181210	*Corynebacterium argentoratense*	Pos	1727	99	99	Human dental plaque	Aerobic	2
DR67-5	20171210	*Cupriavidus gilardii*	Neg	1735	99%	99%	Human dental plaque	Aerobic	1
40	20181210	*Cupriavidus gilardii*	Neg	1731	100	99	Human dental plaque	Aerobic	2
7	20181210	*Elizabethkingia anophelis*	Neg	1494	99	99	Human dental plaque	Aerobic	2
8-3	20181210	*Elizabethkingia anophelis*	Neg	1694	99	99	Human dental plaque	Aerobic	2
11	20181210	*Elizabethkingia anophelis*	Neg	1690	99	99	Human dental plaque	Aerobic	2
14-1	20181210	*Elizabethkingia anophelis*	Neg	1742	100	99	Human dental plaque	Aerobic	2
DR-9	20171110	*Enterobacter hormaechei*	Neg	1727	98%	100%	Human dental plaque	Aerobic	1
DR-10	20171110	*Enterobacter hormaechei*	Neg	1705	99%	99%	Human dental plaque	Aerobic	1
DR-11	20171110	*Enterobacter hormaechei*	Neg	1709	98%	99%	Human dental plaque	Aerobic	1
DR74-1	20171210	*Escherichia coli*	Neg	1426	97%	97%	Human dental plaque	Aerobic	1
DR100-1	20171218	*Escherichia coli*	Neg	2514	100%	100%	Human dental plaque	Aerobic	1
DR52	20171202	*Granulicatella adiacens*	Pos	1243	92%	94%	Human dental plaque	Aerobic	1
DR95-2	20171213	*Klebsiella pneumoniae subsp*	Neg	2492	100%	99%	Human dental plaque	Aerobic	1
DR40-1	20171202	*Lactobacillus casei*	Pos	2343	100%	99%	Human dental plaque	Aerobic	1
DR39-2	20171202	*Lactobacillus casei*	Pos	1456	99%	98%	Human dental plaque	Aerobic	1
DR57-1	20171202	*Lactobacillus casei*	Pos	2591	100%	99%	Human dental plaque	Aerobic	1
DR71-1	20171210	*Lactobacillus casei*	Pos	2555	100%	99%	Human dental plaque	Aerobic	1
DR81-1	20171210	*Lactobacillus casei*	Pos	2593	100%	99%	Human dental plaque	Aerobic	1
DR81-2	20171210	*Lactobacillus casei*	Pos	2628	100%	99%	Human dental plaque	Aerobic	1
DR100-2	20171218	*Lactobacillus casei*	Pos	2593	100%	99%	Human dental plaque	Aerobic	1
DR105-1	20171218	*Lactobacillus casei*	Pos	2588	99%	99%	Human dental plaque	Aerobic	1
DR105-2	20171218	*Lactobacillus casei*	Pos	2601	100%	99%	Human dental plaque	Aerobic	1
DR105-3	20171218	*Lactobacillus casei*	Pos	2573	100%	99%	Human dental plaque	Aerobic	1
DR35-2	20171202	*Lactobacillus casei*	Pos	1727	100%	99%	Human dental plaque	Aerobic	1
DR57-2	20171202	*Lactobacillus casei/paracasei*	Pos	2590	100%	99%	Human dental plaque	Aerobic	1
DR101-2	20171218	*Lactobacillus casei/paracasei*	Pos	2584	100%	99%	Human dental plaque	Aerobic	1
DR48	20171202	*Lactobacillus paracasei*	Pos	2597	100%	100%	Human dental plaque	Aerobic	1
D-2	20181210	*Lactobacillus reuteri*	Pos	1428	97	96	Human dental plaque	Aerobic	2
DR42-3	20171202	*Lactobacillus rhamnosus*	Pos	2662	99%	99%	Human dental plaque	Aerobic	1
DR43-1	20171202	*Lactobacillus rhamnosus*	Pos	2604	100%	99%	Human dental plaque	Aerobic	1
DR43-2	20171202	*Lactobacillus rhamnosus*	Pos	2540	100%	99%	Human dental plaque	Aerobic	1
DR44-1	20171202	*Lactobacillus rhamnosus*	Pos	2597	100%	100%	Human dental plaque	Aerobic	1
DR44-2	20171202	*Lactobacillus rhamnosus*	Pos	2614	100%	100%	Human dental plaque	Aerobic	1
DR71-2	20171210	*Lactobacillus rhamnosus*	Pos	2562	100%	100%	Human dental plaque	Aerobic	1
DR82-1	20171210	*Lactobacillus rhamnosus*	Pos	2612	100%	100%	Human dental plaque	Aerobic	1
DR82-2	20171210	*Lactobacillus rhamnosus*	Pos	2582	100%	100%	Human dental plaque	Aerobic	1
DR82-3	20171210	*Lactobacillus rhamnosus*	Pos	2586	100%	100%	Human dental plaque	Aerobic	1
DR87-1	20171213	*Lactobacillus rhamnosus*	Pos	2630	100%	99%	Human dental plaque	Aerobic	1
DR87-3	20171213	*Lactobacillus rhamnosus*	Pos	2671	99%	99%	Human dental plaque	Aerobic	1
DR87-5	20171213	*Lactobacillus rhamnosus*	Pos	2595	100%	99%	Human dental plaque	Aerobic	1
DR72-2	20171210	*Leifsonia shinshuensis*	Pos	2468	100%	99%	Human dental plaque	Aerobic	1
DR96-3	20171213	*Leifsonia shinshuensis*	Pos	2494	100%	99%	Human dental plaque	Aerobic	1

DR96-4	20171213	*Leifsonia shinshuensis*	Pos	2475	100%	99%	Human dental plaque	Aerobic	1
DR-15	20171115	*Leuconostoc citreum*	Pos	1716	99%	99%	Human dental plaque	Aerobic	1
53-2	20181210	*Leuconostoc citreum*	Pos	1650	100	98	Human dental plaque	Aerobic	2
23	20181210	*Leuconostoc citreum*	Pos	1653	100	98	Human dental plaque	Aerobic	2
53-1	20181210	*Leuconostoc citreum*	Pos	2542	100	99	Human dental plaque	Aerobic	2
DR65-1	20171210	*Leuconostoc lactis*	Pos	2571	100%	99%	Human dental plaque	Aerobic	1
DR75	20171210	*Leuconostoc lactis*	Pos	2558	100%	99%	Human dental plaque	Aerobic	1
DR53-2	20171202	*Microbacterium aurum*	Pos	1546	98%	99%	Human dental plaque	Aerobic	1
DR65-2	20171210	*Microbacterium aurum*	Pos	1688	98%	99%	Human dental plaque	Aerobic	1
DR65-3	20171210	*Microbacterium aurum*	Pos	2505	100%	99%	Human dental plaque	Aerobic	1
DR65-4	20171210	*Microbacterium aurum*	Pos	1441	96%	91%	Human dental plaque	Aerobic	1
DR95-4	20171213	*Microbacterium aurum*	Pos	1729	99%	99%	Human dental plaque	Aerobic	1
B	20181210	*Microbacterium aurum*	Pos	1637	98	97	Human dental plaque	Aerobic	2
DR68	20171210	*Microbacterium lacticum*	Pos	2490	100%	100%	Human dental plaque	Aerobic	1
DR42-4	20171202	*Microbacterium neimengense*	Pos	2494	100%	99%	Human dental plaque	Aerobic	1
DR92-4	20171213	*Microbacterium neimengense*	Pos	2490	100%	99%	Human dental plaque	Aerobic	1
DR67-2	20171210	*Microbacterium paraoxydans*	Pos	2495	100%	100%	Human dental plaque	Aerobic	1
DR73-2	20171210	*Microbacterium resistens*	Pos	2499	100%	99%	Human dental plaque	Aerobic	1
DR73-3	20171210	*Microbacterium resistens*	Pos	2505	100%	99%	Human dental plaque	Aerobic	1
DR106-1	20171218	*Microbacterium resistens*	Pos	2508	100%	100%	Human dental plaque	Aerobic	1
DR106-2	20171218	*Microbacterium resistens*	Pos	2505	99%	99%	Human dental plaque	Aerobic	1
DR-19	20171115	*Microbacterium sp*	Pos	1434	97%	97%	Human dental plaque	Aerobic	1
DR-27	20171115	*Microbacterium sp*	Pos	1735	99%	99%	Human dental plaque	Aerobic	1
DR96-1	20171213	*Microbacterium sp*	Pos	1511	98%	99%	Human dental plaque	Aerobic	1
DR102	20171218	*Microbacterium sp*	Pos	2518	100%	100%	Human dental plaque	Aerobic	1
DR72-1	20171210	*Microbacterium testaceum*	Pos	2471	100%	99%	Human dental plaque	Aerobic	1
52-1	20181210	*Microbacterium trichotecenolyticum*	Pos	1722	100	99	Human dental plaque	Aerobic	2
DR62	20171210	*Nocardia farcinica*	Pos	2495	99%	99%	Human dental plaque	Aerobic	1
45-2	20181210	*Nocardia farcinica*	Pos	1633	98	98	Human dental plaque	Aerobic	2
DR85-2	20171213	*Pediococcus pentosaceus*	Pos	2597	100%	99%	Human dental plaque	Aerobic	1
DR-13	20171115	*Pseudomonas sp.*	Neg	2345	95%	96%	Human dental plaque	Aerobic	1
DR-20	20171115	*Pseudomonas sp.*	Neg	2245	99%	100%	Human dental plaque	Aerobic	1
DR-23	20171115	*Pseudomonas sp.*	Neg	2413	96%	95%	Human dental plaque	Aerobic	1
DR96-2	20171213	*Roseomonas gilardii*	Neg	2436	100%	99%	Human dental plaque	Aerobic	1
DR36-3	20171202	*Rothia mucilaginosa*	Pos	2449	100%	99%	Human dental plaque	Aerobic	1
DR36-4	20171202	*Rothia mucilaginosa*	Pos	2507	100%	99%	Human dental plaque	Aerobic	1
DR42-1	20171202	*Rothia mucilaginosa*	Pos	2468	100%	99%	Human dental plaque	Aerobic	1
DR42-2	20171202	*Rothia mucilaginosa*	Pos	2444	100%	99%	Human dental plaque	Aerobic	1
DR46-2	20171202	*Rothia mucilaginosa*	Pos	1729	99%	99%	Human dental plaque	Aerobic	1
DR58-1	20171202	*Rothia mucilaginosa*	Pos	2470	99%	99%	Human dental plaque	Aerobic	1
DR63-2	20171210	*Rothia mucilaginosa*	Pos	1736	99%	99%	Human dental plaque	Aerobic	1
DR64-1	20171210	*Rothia mucilaginosa*	Pos	1445	96%	98%	Human dental plaque	Aerobic	1
DR64-2	20171210	*Rothia mucilaginosa*	Pos	1733	99%	99%	Human dental plaque	Aerobic	1
DR64-3	20171210	*Rothia mucilaginosa*	Pos	1722	99%	99%	Human dental plaque	Aerobic	1
DR76	20171210	*Rothia mucilaginosa*	Pos	1725	99%	99%	Human dental plaque	Aerobic	1
DR77-2	20171210	*Rothia mucilaginosa*	Pos	1722	99%	99%	Human dental plaque	Aerobic	1
DR86	20171213	*Rothia mucilaginosa*	Pos	2479	100%	99%	Human dental plaque	Aerobic	1
DR87-4	20171213	*Rothia mucilaginosa*	Pos	2455	100%	99%	Human dental plaque	Aerobic	1
DR88	20171213	*Rothia mucilaginosa*	Pos	2505	100%	99%	Human dental plaque	Aerobic	1
DR90	20171213	*Rothia mucilaginosa*	Pos	1731	99%	99%	Human dental plaque	Aerobic	1
DR92-3	20171213	*Rothia mucilaginosa*	Pos	1735	99%	99%	Human dental plaque	Aerobic	1
DR94	20171213	*Rothia mucilaginosa*	Pos	1317	94%	95%	Human dental plaque	Aerobic	1
9-2	20181210	*Rothia mucilaginosa*	Pos	1711	100	99	Human dental plaque	Aerobic	2
9-3	20181210	*Rothia mucilaginosa*	Pos	1637	98	98	Human dental plaque	Aerobic	2
15-1	20181210	*Rothia mucilaginosa*	Pos	1701	100	99	Human dental plaque	Aerobic	2
18	20181210	*Rothia mucilaginosa*	Pos	2486	100	99	Human dental plaque	Aerobic	2
59-1	20181210	*Rothia mucilaginosa*	Pos	1703	99	99	Human dental plaque	Aerobic	2
59-2	20181210	*Rothia mucilaginosa*	Pos	1714	99	99	Human dental plaque	Aerobic	2
61-1	20181210	*Rothia mucilaginosa*	Pos	1711	100	99	Human dental plaque	Aerobic	2
DR41-2	20171202	*Staphylococcus epidermidis*	Pos	1733	99%	100%	Human dental plaque	Aerobic	1
DR41-3	20171202	*Staphylococcus epidermidis*	Pos	1722	99%	99%	Human dental plaque	Aerobic	1
DR49	20171202	*Staphylococcus epidermidis*	Pos	1738	99%	99%	Human dental plaque	Aerobic	1
DR50-1	20171202	*Staphylococcus epidermidis*	Pos	1731	99%	99%	Human dental plaque	Aerobic	1
DR50-2	20171202	*Staphylococcus epidermidis*	Pos	1731	99%	99%	Human dental plaque	Aerobic	1
DR54-1	20171202	*Staphylococcus epidermidis*	Pos	1463	99%	98%	Human dental plaque	Aerobic	1
DR54-2	20171202	*Staphylococcus epidermidis*	Pos	1522	98%	99%	Human dental plaque	Aerobic	1
DR60-1	20171210	*Staphylococcus epidermidis*	Pos	1461	99%	97%	Human dental plaque	Aerobic	1
DR60-2	20171210	*Staphylococcus epidermidis*	Pos	1509	98%	99%	Human dental plaque	Aerobic	1
DR61-2	20171210	*Staphylococcus epidermidis*	Pos	2627	99%	100%	Human dental plaque	Aerobic	1
DR63-1	20171210	*Staphylococcus epidermidis*	Pos	1712	99%	99%	Human dental plaque	Aerobic	1
DR66	20171210	*Staphylococcus epidermidis*	Pos	1465	99%	98%	Human dental plaque	Aerobic	1
DR74-3	20171210	*Staphylococcus epidermidis*	Pos	1489	99%	98%	Human dental plaque	Aerobic	1
DR74-4	20171210	*Staphylococcus epidermidis*	Pos	1480	99%	98%	Human dental plaque	Aerobic	1
DR77-1	20171210	*Staphylococcus epidermidis*	Pos	1482	99%	98%	Human dental plaque	Aerobic	1
DR77-3	20171210	*Staphylococcus epidermidis*	Pos	1500	99%	98%	Human dental plaque	Aerobic	1
DR80	20171210	*Staphylococcus epidermidis*	Pos	1718	99%	99%	Human dental plaque	Aerobic	1
DR82-4	20171213	*Staphylococcus epidermidis*	Pos	1439	99%	97%	Human dental plaque	Aerobic	1
DR84-1	20171213	*Staphylococcus epidermidis*	Pos	2555	100%	100%	Human dental plaque	Aerobic	1
DR84-2	20171213	*Staphylococcus epidermidis*	Pos	2560	100%	99%	Human dental plaque	Aerobic	1
DR85-1	20171213	*Staphylococcus epidermidis*	Pos	2526	100%	100%	Human dental plaque	Aerobic	1
DR89-2	20171213	*Staphylococcus epidermidis*	Pos	2555	100%	100%	Human dental plaque	Aerobic	1
DR89-4	20171213	*Staphylococcus epidermidis*	Pos	2577	100%	99%	Human dental plaque	Aerobic	1
DR91-1	20171213	*Staphylococcus epidermidis*	Pos	1517	99%	98%	Human dental plaque	Aerobic	1
DR91-2	20171213	*Staphylococcus epidermidis*	Pos	1543	99%	99%	Human dental plaque	Aerobic	1
DR93-1	20171213	*Staphylococcus epidermidis*	Pos	1504	99%	99%	Human dental plaque	Aerobic	1
DR93-2	20171213	*Staphylococcus epidermidis*	Pos	1537	98%	99%	Human dental plaque	Aerobic	1
24-1	20181210	*Staphylococcus epidermidis*	Pos	1166	97	99	Human dental plaque	Aerobic	2
1-1	20181210	*Staphylococcus epidermidis*	Pos	1692	99	99	Human dental plaque	Aerobic	2
1-4	20181210	*Staphylococcus epidermidis*	Pos	1712	100	99	Human dental plaque	Aerobic	2
3-1	20181210	*Staphylococcus epidermidis*	Pos	1720	100	99	Human dental plaque	Aerobic	2
3-2	20181210	*Staphylococcus epidermidis*	Pos	1729	100	99	Human dental plaque	Aerobic	2
4-4-1	20181210	*Staphylococcus epidermidis*	Pos	1727	100	99	Human dental plaque	Aerobic	2
4-5	20181210	*Staphylococcus epidermidis*	Pos	1696	99	99	Human dental plaque	Aerobic	2

20	20181210	*Staphylococcus epidermidis*	Pos	1709	99	99	Human dental plaque	Aerobic	2
4-4-2	20181210	*Staphylococcus haemolyticus*	Pos	1716	100	99	Human dental plaque	Aerobic	2
5-3	20181210	*Staphylococcus haemolyticus*	Pos	1701	100	99	Human dental plaque	Aerobic	2
5-5	20181210	*Staphylococcus haemolyticus*	Pos	1666	99	99	Human dental plaque	Aerobic	2
DR91-3	20171213	*Staphylococcus saprophyticus*	Pos	1738	99%	99%	Human dental plaque	Aerobic	1
DR97-1	20171213	*Staphylococcus saprophyticus*	Pos	2488	100%	100%	Human dental plaque	Aerobic	1
DR89-5	20171213	*Staphylococcus saprophyticus*	Pos	2564	100%	99%	Human dental plaque	Aerobic	1
DR89-6	20171213	*Staphylococcus saprophyticus*	Pos	2564	100%	99%	Human dental plaque	Aerobic	1
DR92-1	20171213	*Staphylococcus saprophyticus*	Pos	2555	100%	100%	Human dental plaque	Aerobic	1
DR92-2	20171213	*Staphylococcus saprophyticus*	Pos	2555	100%	100%	Human dental plaque	Aerobic	1
DR97-2	20171213	*Staphylococcus saprophyticus*	Pos	1317	98%	95%	Human dental plaque	Aerobic	1
DR-1	20171101	*Stenotrophomonas maltophilia*	Neg	1709	99%	99%	Human dental plaque	Aerobic	1
DR-3	20171101	*Stenotrophomonas maltophilia*	Neg	1738	99%	100%	Human dental plaque	Aerobic	1
DR-4	20171101	*Stenotrophomonas maltophilia*	Neg	2516	100%	99%	Human dental plaque	Aerobic	1
DR-5	20171101	*Stenotrophomonas maltophilia*	Neg	1725	99%	99%	Human dental plaque	Aerobic	1
DR-6	20171030	*Stenotrophomonas maltophilia*	Neg	1729	99%	99%	Human dental plaque	Aerobic	1
DR73-1	20171210	*Stenotrophomonas maltophilia*	Neg	2498	100%	99%	Human dental plaque	Aerobic	1
DR73-4	20171210	*Stenotrophomonas maltophilia*	Neg	1522	99%	99%	Human dental plaque	Aerobic	1
DR99	20171218	*Stenotrophomonas maltophilia*	Neg	2505	99%	99%	Human dental plaque	Aerobic	1
DR-28	20171115	*Stenotrophomonas maltophilia*	Neg	1709	99%	99%	Human dental plaque	Aerobic	1
DR-29	20171115	*Stenotrophomonas maltophilia*	Neg	1738	99%	99%	Human dental plaque	Aerobic	1
DR-33	20171115	*Stenotrophomonas maltophilia*	Neg	2516	99%	99%	Human dental plaque	Aerobic	1
DR-34	20171115	*Stenotrophomonas maltophilia*	Neg	1725	99%	100%	Human dental plaque	Aerobic	1
5-1	20181210	*Stenotrophomonas maltophilia*	Neg	1729	100	99	Human dental plaque	Aerobic	2
6-1	20181210	*Stenotrophomonas maltophilia*	Neg	1712	99	99	Human dental plaque	Aerobic	2
8-2	20181210	*Stenotrophomonas maltophilia*	Neg	1712	100	99	Human dental plaque	Aerobic	2
30-2	20181210	*Stenotrophomonas maltophilia*	Neg	1742	100	99	Human dental plaque	Aerobic	2
38	20181210	*Stenotrophomonas maltophilia*	Neg	1718	100	99	Human dental plaque	Aerobic	2
45-1	20181210	*Stenotrophomonas maltophilia*	Neg	1716	99	99	Human dental plaque	Aerobic	2
48	20181210	*Stenotrophomonas maltophilia*	Neg	1717	100	99	Human dental plaque	Aerobic	2
D-1	20181210	*Stenotrophomonas maltophilia*	Neg	1709	100	99	Human dental plaque	Aerobic	2
DR36-1	20171202	*Streptococcus mitis*	Pos	2514	100%	99%	Human dental plaque	Aerobic	1
DR37-1	20171202	*Streptococcus mitis*	Pos	2462	100%	99%	Human dental plaque	Aerobic	1
DR37-2	20171202	*Streptococcus mitis*	Pos	2475	100%	99%	Human dental plaque	Aerobic	1
DR37-3	20171202	*Streptococcus mitis*	Pos	1696	99%	99%	Human dental plaque	Aerobic	1
56	20181210	*Streptococcus mitis*	Pos	1712	99	99	Human dental plaque	Aerobic	2
55-1	20181210	*Streptococcus parasanguinis*	Pos	1646	99	98	Human dental plaque	Aerobic	2
DR55-1	20171202	*Streptococcus pseudopneumoniae*	Pos	2553	100%	99%	Human dental plaque	Aerobic	1
DR55-2	20171202	*Streptococcus pseudopneumoniae*	Pos	2555	100%	99%	Human dental plaque	Aerobic	1
DR67-4	20171210	*Tsukamurella inchonensis*	Pos	2483	100%	99%	Human dental plaque	Aerobic	1
DR38-2	20171202	*Weissella confusa*	Pos	2603	100%	99%	Human dental plaque	Aerobic	1
DR38-3	20171202	*Weissella confusa*	Pos	2569	100%	100%	Human dental plaque	Aerobic	1
DR79-1	20171210	*Weissella confusa*	Pos	1208	95%	94%	Human dental plaque	Aerobic	1
